# Patients aged 80 years or older with non-ST-elevation myocardial infarction or unstable angina pectoris randomised to an invasive versus conservative strategy: angiographic and procedural results from the After Eighty study

**DOI:** 10.1136/openhrt-2020-001256

**Published:** 2020-07-22

**Authors:** Nicolai Tegn, Christian Eek, Michael Abdelnoor, Lars Aaberge, Knut Endresen, Rita Skårdal, Erlend Sturle Berg, Lars Gullestad, Bjørn Bendz

**Affiliations:** 1 Department of Cardiology, Oslo University Hospital, Rikshospitalet, Oslo, Norway; 2 Institute of Clinical Medicine, Faculty of Medicine, University of Oslo, Oslo, Norway

**Keywords:** coronary artery disease, atherosclerosis, risk factors

## Abstract

**Objectives:**

We aimed to report the angiographic and procedural results of the After Eighty study (ClinicalTrials.gov, NCT01255540), and to identify independent predictors of revascularisation.

**Methods:**

Patients of ≥80 years old with non-ST-elevation myocardial infarction and unstable angina pectoris were randomised to an invasive or conservative strategy. Angiographic and procedural results were recorded. Univariate and multivariate analyses were performed to explore variables predicting revascularisation.

**Results:**

Among 229 patients in the invasive group, 220 underwent immediate coronary angiography (90% performed via the radial artery). Of these patients, 48% had three-vessel disease or left main stenosis, 18% two-vessel disease, 16% one-vessel disease, 17% minor coronary vessel wall changes and two patients had normal coronary arteries. Six patients (3%) underwent coronary artery bypass graft. Percutaneous coronary intervention (PCI) was performed in 107 patients (49%), with 57% treated with bare metal stents, 37% drug-eluting stents and 6% balloon angioplasty. On average, 1.7 lesions were treated and 2 stents delivered per patient. Complications included 1 major PCI-related bleeding (successfully treated), 2 minor access site-related bleedings, 3 side branch occlusions during PCI and 11 periprocedural myocardial infarctions (considered end points). Sex, bundle branch block and smoking were independent predictors of revascularisation.

**Conclusions:**

PCI was performed in approximately half of the patients, similar to findings in younger populations. Procedural success was high, with few complications.

**Trial registration number:**

NCT01255540

**Key questions**

**What is already known about this subject?**

- The After Eighty study was the first randomised controlled trial specifically targeting very elderly patients with non-ST-elevation myocardial infarction (NSTEMI) or unstable angina pectoris (UAP). The results indicated that the invasive strategy was superior to the conservative strategy, and that bleeding complication rates were similar between the two groups.

**What does this study add?**

- Previous trials of considerably younger patients have not shared angiographic and procedural data. In the After Eighty study, procedural success was high and complications few despite a high proportion of the patients having three-vessel and/or left main disease.

**How might this impact on clinical practice?**

- The trial demonstrates that invasive management of NSTEMI or UAP can be performed in clinically stable octogenarians without compromising patient safety.

## Introduction

Ischaemic heart disease is the leading cause of death among patients in the USA and Europe.^1^ Cardiovascular disease is particularly common among very elderly individuals, that is, those aged 80 years or older. As this age group grows, the absolute prevalence of cardiovascular disease is expected to increase further.^2^

In the After Eighty study, patients aged 80 years or older who presented with non-ST-elevation myocardial infarction (NSTEMI) and unstable angina pectoris (UAP) were randomised to management using either an invasive or conservative approach.^3^ The primary end point was a composite of myocardial infarction, required urgent revascularisation, stroke or death. The results indicated that the invasive strategy was superior to the conservative strategy, and that bleeding complication rates were similar between the two groups. The superiority of the invasive strategy was diluted with increasing age, and was not reflected by changes in health-related quality of life, as measured by the SF-36.^4^

Here, we report the angiographic and procedural results of the After Eighty study. Analysis was performed to identify independent predictors of revascularisation.

## Methods

The After Eighty study (ClinicalTrials.gov number NCT01255540) was an open-label, prospective, randomised, controlled, multicentre trial. It included 457 patients admitted to 16 hospitals without percutaneous coronary intervention (PCI) facilities in the South-East Health Region of Norway between 2010 and 2014. Consecutive patients were evaluated for study eligibility within 2 days after hospital admission year round. Those fulfilling the inclusion criteria were invited to participate. All participants gave their written informed consent before inclusion. The protocol was approved by the relevant institutional review boards and the regional board of research ethics.

Enrolled patients were randomised to either an invasive or a conservative management strategy. The conservative strategy involved optimal medical therapy (OMT) administered at the community hospital. The invasive strategy involved early coronary angiography with immediate evaluation for ad hoc PCI, coronary artery bypass graft (CABG) or OMT. Patients randomised to the invasive strategy were transported to Oslo University Hospital, and underwent coronary angiography 1 day after inclusion. Patients who underwent only coronary angiography were returned to their local hospital after 4–6 hours. Those requiring PCI were returned after 6–18 hours, depending on the treated segments and the travel distance. The primary end point was a composite of myocardial infarction, required urgent revascularisation, stroke and death.

The angiographic and procedural methods were in accordance with generally accepted guidelines and routines involving digital imaging acquisition and storage.^5 6^ The radial artery was the preferred access approach. Lesions were imaged in at least two different projections, preferably at 90°, especially for eccentric stenoses. A lesion was deemed significant when it reduced luminal diameter by at least 50% (ie, 75% area stenosis) based on visual assessment. The culprit lesion was identified using a combination of ECG, echocardiographic and angiographic findings. Fractional flow reserve, optical coherence tomography (OCT) and intravascular ultrasound (IVUS) were not routinely performed.

The SYNTAX score was used to grade coronary anatomy, and to guide patient selection for optimal revascularisation treatment.^7^ The residual SYNTAX score was calculated to quantify revascularisation completeness. Before deciding the revascularisation strategy for each patient, coronary angiograms were reviewed to consensus by at least two invasive cardiologists. Including the process for research data interpretation, the coronary angiograms were ultimately evaluated by four independent invasive cardiologists before consensus was made.

When patients in the conservative group experienced reinfarction, refractory angina pectoris, malignant ventricular arrhythmias or increasing heart failure symptoms, they were considered for urgent coronary angiography by doctors at the community hospitals (considered as end point). The low viscosity and non-ionic iodixanol (Visipaque 320, GE Healthcare) were used as contrast medium. Contrast-induced nephropathy (CIN) was defined as renal function impairment measured as a 25% increase in serum creatinine within 48–72 hours after contrast administration.

### Statistical analysis

All values are presented as mean and SD unless otherwise stated. We performed univariate and multivariate analyses, using revascularisation as the end point, to identify predictors of revascularisation in the intervention group. Univariate analysis was performed using contingency tables for dichotomised variables, and the Mann-Witney test for the continuous variables for patients with or without revascularisation. Multivariate analysis was performed using a logistic model to identify independent predictors of revascularisation. To assess the model’s predictive accuracy, we evaluated the calibration using the Hosmer and Lemeshow goodness-of-fit test, and the discrimination via analysis of the area under the Receiver Operating Characteristic (ROC) curve. An area under the curve of >0.7 indicates that a model has acceptable discriminatory capability.^8^ This substudy was underpowered to identify predictors of mortality and reinfarction.

## Results

Tables 1 and 2 present the patients’ baseline characteristics and medical treatment. Among the 457 patients in the After Eighty study, 229 were randomised to the invasive strategy. Within 24 hours after randomisation, five patients in the invasive group dropped out of the study.^3^ Another four patients did not undergo coronary angiography due to stroke (n=1), gastrointestinal bleedings (n=2) and refractory delirium (n=1) shortly after randomisation. Consequently, 220 patients in the invasive group underwent immediate coronary angiography. None of the 228 patients in the conservative group underwent immediate coronary angiography. However, 56 patients in the conservative group were eventually referred for coronary angiography due to need for revascularisation or acute coronary syndrome.

**Table 1:** Baseline characteristics

Characteristic	Invasive strategy (n=220)
Age in years, mean (range)	84.7 (80–93)
Male, n (%)	122 (55)
Medical history, n (%)	
Previous myocardial infarction	103 (47)
Previous angina	122 (55)
Previous PCI	53 (24)
Previous CABG	42 (19)
Hypertension	127 (58)
Type 2 diabetes	45 (20)
Chronic obstructive pulmonary disease	23 (10)
Apoplexia cerebri	38 (17)
Peripheral vascular disease	19 (9)
Atrial fibrillation	47 (21)
Smoking status, n (%)	
Current	17 (8)
Previous	93 (42)
EF, n (%)	
EF <30%	11 (5)
EF 30%–50%	60 (27)
EF >50%	103 (47)
ECG at admission, n (%)	
Atrial fibrillation	49 (22)
Pathological Q-wave	35 (16)
ST depression	40 (18)
Negative T-wave	31 (14)
Right bundle branch block	21 (10)
Left bundle branch block	21 (10)
Troponin elevation*	210 (95)
Creatinine, μmol/L	102.4

Values are mean±SD unless otherwise indicated.

*Troponin levels exceeding the 99th percentile of a normal population.

CABG, coronary artery bypass graft; EF, left ventricular ejection fraction; PCI, percutaneous coronary intervention.

**Table 2:** Medical treatment at discharge

Characteristic	Invasive strategy (n=220)
Medical therapy at discharge, n (%)	
Acetylsalicylic acid	211 (96)
Clopidogrel	162 (74)
Ticagrelor	8 (4)
Warfarin	47 (21)
Dabigatran	1 (0.5)
Rivaroxaban	3 (1)
Beta blocker	188 (85)
Statins	203 (92)
ACE inhibitor/ARB	117 (53)
Calcium channel blocker	53 (24)
Nitrates	76 (35)

ACE, angiotensin-converting enzyme; ARB, angiotensin receptor blocker.

Of the coronary angiographies, 90% were performed via the radial artery. Among the patients in the invasive group, 48% had three-vessel disease or left main stenosis, 18% had two-vessel disease, 16% had one-vessel disease, 17% had minor coronary vessel wall changes and two patients had normal coronary arteries. Thus, of the 220 patients, 180 (82%) had obstructive coronary disease. Eleven patients (5%) had an acute occlusive thrombotic lesion. CABG was performed in six patients (3%). PCI was performed in 107 patients (49%), of whom 57% were treated with bare metal stents (BMS), 37% with drug-eluting stents (DES) and 6% with balloon angioplasty alone. In each patient, an average of 1.7 lesions were treated, and 2 stents were delivered. The median Syntax score was 12 (range, 0–66), with 69% having a score of ≤22, 18% a score of 23–32 and 13% a score of >32.

Patients who underwent angiography alone received a median of 65 mL contrast (range, 35–150 mL). Patients treated with ad hoc PCI received a median of 180 mL contrast (range, 80–515 mL). No serious ventricular arrhythmias were registered during the procedures. Table 3 presents additional angiographic data.

**Table 3:** Details regarding coronary angiography and intervention, at index and reintervention, in the invasive strategy group

Characteristic	Invasive strategy (n=220)
Days from inclusion to angiography	1.4
Coronary angiographic and interventional data at index event, n (%)	
Three-vessel disease or left main	105 (48)
Two-vessel disease	40 (18)
One-vessel disease	35 (16)
Calcification, no significant stenosis	38 (17)
Normal	2 (1)
No previous CABG, n (%)	
Left main lesion	17 (8)
LAD lesion	138 (63)
CX lesion	115 (52)
RCA lesion	115 (52)
Previous CABG, n (%)	41 (19)
Left main lesion	19 (46)
LAD lesion	40 (98)
CX lesion	37 (90)
RCA lesion	36 (88)
Occluded graft/LIMA	17/4
Treatment	
PCI	107 (49)
BMS (% of total PCI)	61 (57)
DES (% of total PCI)	40 (37)
POBA (% of total PCI)	6 (6)
CABG	6 (3)
Medical treatment only	107 (49)
Chronic total occlusion	74
Calcified lesion	95
Thrombotic lesion	11
No. treated lesions per patient	1.7
No. stents implanted per patient	2
Total stent length per patient in mm	35
Receipt of allocated stent type	100%
Segments with angiographic success	87%
Syntax score, median (range)	12 (0–66)
Residual Syntax score, median (range)	1 (0–66)
Complications	
Occluded side branch, %	3
Perforation	1
Radial/femoral access, n (%)	198 (90)
Contrast (angiography) in mL, median (range)	65 (35–150)
Contrast (angiography+PCI) in mL, median (range)	180 (80–515)
Coronary angiographic data and interventional data after readmission, n (%)	
Number	12 (5)
Target lesion	3
Stent thrombosis	0
New lesion	4
PCI	7
No intervention	5

BMS, bare metal stent; CABG, coronary artery bypass graft; CX, ramus circumflexus; DES, drug-eluting stent; LAD, left anterior descending artery; PCI, percutaneous coronary intervention; POBA, plain old balloon angioplasty; RCA, right coronary artery.

In the conservative group, 56 patients (25%) were referred for coronary angiography. The median time to angiography was 38 days (range, 1–1160 days) after randomisation. Of these patients, 33 (59%) subsequently underwent PCI. Table 4 presents additional angiographic data.

**Table 4:** Details regarding coronary angiography and intervention in the conservative group

Characteristic	Conservative (n=56)
Days from inclusion to angiography	130
Coronary angiographic and interventional data at index event, n (%)	
Three-vessel disease or left main	19 (34)
Two-vessel disease	20 (36)
One-vessel disease	13 (23)
Calcification, no significant stenosis	4 (7)
Normal	0 (0)
No previous CABG	51
Left main lesion	9 (18)
LAD lesion	35 (67)
CX lesion	29 (57)
RCA lesion	27 (53)
Previous CABG	5
Left main lesion	4 (80)
LAD lesion	5 (100)
CX lesion	5 (100)
RCA lesion	5 (100)
Occluded graft/LIMA	4/0
Treatment	
PCI	33 (59)
BMS (% of total PCI)	13 (39)
DES (% of total PCI)	19 (58)
POBA (% of total PCI)	1 (3)
CABG	0 (0)
Medical treatment only	23 (41)
Chronic total occlusion	22
No. of treated lesions per patient	1.6
No. of stents implanted per patient	2.3
Total stent length per patient in mm	38
Receipt of allocated stent type	100%

BMS, bare metal stent; CABG, coronary artery bypass graft; CX, ramus circumflexus; DES, drug-eluting stent; LAD, left anterior descending artery; LIMA, Left Internal Mammary Artery; PCI, percutaneous coronary intervention; POBA, plain old balloon angioplasty; RCA, right coronary artery.

Univariate analysis using contingency tables revealed that the incidence of revascularisation was lower among women (39.2%) than men (62.2%) (p<0.01). The incidence of revascularisation was lower among patients with left bundle branch block (LBBB) (16.1%) compared with the patients with right bundle branch block (60%) and no bundle branch block (54%; p=0.03). Current smokers had a lower incidence of revascularisation (23.5%) compared with previous smokers (58.6%) and non-smokers (48.4%) (p=0.02).

Multivariate analysis using a logistic model confirmed the three variables sex, bundle branch block and smoking to be significant independent predictors of revascularisation. The model showed acceptable discrimination (0.68) and satisfactory calibration (p=0.31). Women had 65% less revascularisation than men (p=0.0001). Current smokers had a 77% lower incidence of revascularisation compared with previous smokers and non-smokers (p=0.02). Patients with LBBB had a 54% lower incidence of revascularisation compared with those with right bundle branch block and no bundle branch block (p=0.05).

Previous angina pectoris, CABG, atrial fibrillation, hypertension, chronic obstructive pulmonary disease and type 2 diabetes were not predictors of coronary intervention.

### Complications

One incident of major bleeding (pericardial tamponade) occurred in relation to the PCI procedure and was successfully treated. Two minor bleeding complications occurred in relation to the access site (arteria radialis). Three patients experienced side branch occlusion during PCI. A total of 11 periprocedural myocardial infarctions occurred (considered as end points). No procedural stroke was observed, and Syntax score did not predict procedural complications.

The mean overall hospital stay length was 6 days in the invasive group, and 5 days in the conservative group. A creatinine elevation of >25% occurred during the index hospital stay in seven patients (3.1%) in the invasive group, and four (1.8%) in the conservative group. Of the seven patients in the invasive group, two (0.9%) had a urinary tract infection. In the remaining five patients (2.2%) patients, CIN could not be excluded as a possible cause of increased creatinine.

## Discussion

Within the invasive strategy arm of the After Eighty study, 180 (82%) of the 220 patients had obstructive coronary disease, while 17% had minor coronary vessel wall changes and 1% had normal coronary arteries. Eleven patients (5%) had an acute occlusive thrombotic lesion. Of these patients, 49% underwent PCI and 3% underwent CABG. Within the conservative study arm, 25% of patients were eventually referred to a coronary angiography at a median of 38 days after randomisation. Of these patients, 59% underwent PCI. The rates of procedural bleeding complications were low: 0.4% major and 1.0% minor. Eleven patients (5%) experienced periprocedural myocardial infarctions.

Patients with NSTEMI and UAP show diverse angiographic patterns of coronary artery disease, ranging from normal epicardial coronary arteries to a severely and diffusely diseased coronary artery tree.^9^ Among very elderly patients, treatment is often complicated by additional challenges, such as complex multivessel coronary calcification disease, tortuous vascular anatomy, impaired ventricular function, a higher risk profile and substantial comorbidity.^10 11^ Comparing the present findings with the results of previous trials is not straightforward. In FRISC II, ICTUS and RITA-3, the median age was under 65 years,^12–14^ and angiographic and procedural data are difficult to obtain (personal communication with steering committees). The Italian Elderly Acute Coronary Syndrome (ACS) study included patients of ≥75 years old,^15^ and its design differed from the present study, specifically regarding the fact that only medically stabilised patients were randomised in the After Eighty study. Within the early aggressive group of the Italian Elderly ACS study, 48% underwent PCI and 6% CABG during the index admission, which is comparable with the rates in our study. The two studies also showed similar lesion distributions.

The median amounts of contrast used—65 mL for diagnostic angiography and 180 mL for angiography plus ad hoc PCI—were greater than expected. Despite this, only five patients (2.2%) exhibited a >25% rise in creatinine during the index hospital stay (6 days). Adequate hydration and the type of contrast media used appear to be critical factors in patients at risk of CIN. When angiography/PCI is performed, patients are generally well hydrated and not in a fasting condition. Use of the low viscosity and non-ionic iodixanol as the contrast medium may also offer renal protection. In the invasive group, 113 patients (51.3%) underwent angiography alone. These factors may have reduced the risk of CIN despite the age of the population. However, CIN can develop up to 1 week after contrast administration; thus, it is possible that we may have failed to detect CIN in some individuals.

Most bleeding was of gastrointestinal origin and was probably due to double antiplatelet therapy. Except for one pericardial tamponade and two minor bleeding complications related to the access site, bleeding complication rates were similar between the two strategy groups. Among elderly patients undergoing cardiac catheterisation, the rates of major bleeding or access site complications are lower with radial access compared with femoral access.^16^ In the After Eighty study, radial access was used in 90% of patients, and only 2 of 198 patients experienced minor access site-related bleeding. Overall, the evidence supports the use of radial access among very elderly patients.

In small studies designed and powered for composite end points, subanalysis results must be interpreted cautiously. Here, we performed a possible hypothesis generating subanalysis to explore whether we could find any possible variables predicting revascularisation, because many clinicians wonder if there are predictors that can help them to choose the right patient at the right strategy. We identified a trend towards lower intervention rates among women, patients with LBBB and smokers. The explanation for these trends remains unclear, and the present study is not powered to determine whether this may be due to non-obstructive coronary artery disease, small vessel disease or very complex lesions. However, one may speculate if the trends are due to small coronary lumen diameter in women, extensive obstructive coronary artery disease in LBBB and the smokers paradox.

Within the invasive arm of this study, approximately half of the patients with significant stenosis did not undergo PCI. The reasons included small vessel disease ineligible for revascularisation therapy, severe coronary artery disease (eg, three-vessel/left main) combined with severe peripheral disease (ineligible for PCI/CABG) and severe coronary artery disease after CABG ineligible for redo surgery and PCI. Moreover, it can be challenging to detect the culprit lesion in acute coronary syndrome. The lesions may have both ruptured or intact fibrous caps and may be difficult to analyse. In the present trial, OCT and IVUS were not routinely used, but would probably have increased the precision. However, OCT and IVUS may complicate the invasive procedure in advanced disease as found in the present population.

Within the conservative strategy arm of this study, 69 patients experienced myocardial infarction and 24 patients required urgent revascularisation (considered end points). Only 56 of these patients were referred for coronary angiography. We believe that this rather conservative approach was due to a lack of evidence of coronary intervention in this group, as well as the advanced age. In both strategy arms, the doctors at the community hospitals decided whether a patient with myocardial infarction or refractory angina pectoris should be scheduled for coronary angiography.

Among the treated patients, 57% received BMS, 37% DES and 6% balloon angioplasty only. During the inclusion period (2010–2014), it was thought that stent thrombosis was more frequent among patients receiving DES than BMS. Dual antiplatelet therapy was mandatory in both the invasive and conservative strategy arms. However, in the event of a serious bleeding complication (which was very likely in this very old population), a single platelet inhibitor was considered safer after implantation of a BMS compared with a DES. The same was believed for patients requiring anticoagulation (eg, atrial fibrillation). However, during the last part of the inclusion period, there was a trend towards more frequent use of DES. It was later shown that the rates of repeat revascularisation and stent thrombosis are lower after DES implantation.^17^

In the present trial, the invasive treatment strategy was decided based on the consensus of at least two invasive cardiologists prior to selection of the revascularisation strategy in each patient. Compared with a full revascularisation strategy, it was considered preferable to treat symptomatic and prognostic lesions (eg, left main and proximal left anterior descending artery), eligible subtotal stenosis and the most likely culprit lesion. This approach was favoured due to the advanced age of the patients. It is unclear whether this strategy is warranted.

In conclusion, the After Eighty study included octogenarians with NSTEMI and UAP who were randomised to an invasive or conservative strategy. A high proportion of patients had three-vessel and/or left main disease. PCI was performed in approximately half of the patients with a high procedural success and with few complications. Sex, bundle branch block and smoking were independent predictors of revascularisation.
